# Accounting for observation processes across multiple levels of uncertainty improves inference of species distributions and guides adaptive sampling of environmental DNA

**DOI:** 10.1002/ece3.4552

**Published:** 2018-10-23

**Authors:** Amy J. Davis, Kelly E. Williams, Nathan P. Snow, Kim M. Pepin, Antoinette J. Piaggio

**Affiliations:** ^1^ United States Department of Agriculture, Animal and Plant Health Inspection Service, Wildlife Services, National Wildlife Research Center Fort Collins Colorado; ^2^ School of Environmental and Forest Sciences University of Washington Seattle Washington

**Keywords:** detection, environmental DNA, invasive species, monitoring, multi‐scale occupancy, *Sus scrofa*

## Abstract

Understanding factors that influence observation processes is critical for accurate assessment of underlying ecological processes. When indirect methods of detection, such as environmental DNA, are used to determine species presence, additional levels of uncertainty from observation processes need to be accounted for. We conducted a field trial to evaluate observation processes of a terrestrial invasive species (wild pigs‐ *Sus scrofa*) from DNA in water bodies. We used a multi‐scale occupancy analysis to estimate different levels of observation processes (detection, *p*): the probability DNA is available per sample (*θ*), the probability of capturing DNA per extraction (*γ*), and the probability of amplification per qPCR run (*δ*). We selected four sites for each of three water body types and collected 10 samples per water body during two months (September and October 2016) in central Texas. Our methodology can be used to guide sampling adaptively to minimize costs while improving inference of species distributions. Using a removal sampling approach was more efficient than pooling samples and was unbiased. Availability of DNA varied by month, was considerably higher when water pH was near neutral, and was higher in ephemeral streams relative to wildlife guzzlers and ponds. To achieve a cumulative detection probability >90% (including availability, capture, and amplification), future studies should collect 20 water samples per site, conduct at least two extractions per sample, and conduct five qPCR replicates per extraction. Accounting for multiple levels of uncertainty of observation processes improved estimation of the ecological processes and provided guidance for future sampling designs.

## 1 INTRODUCTION

A primary challenge to understanding ecological processes and the patterns they produce (e.g., survival, abundance, distribution) is that they are rarely, if ever, observed perfectly. Understanding variation in the ability to detect a target species in the wild is necessary for disentangling the noise in observation processes (i.e., detection) from the signal of ecological processes of interest. The need to account for observation processes is well recognized in ecology, and many factors are known to impact these processes including observer error (Nichols, Hines, Sauer, Fallon, & Heglund, 2000), environmental conditions (Simons, Alldredge, Pollock, & Wettroth, 2007), detection method (Digby, Towsey, Bell, & Teal, 2013), and species behavior (Diefenbach, Marshall, Mattice, & Brauning, 2007). In molecular ecology, there are additional levels of uncertainty in observation processes dealing not just with species availability and human observation error but also with DNA availability and assay specificity and sensitivity (Willoughby, Wijayawardena, Sundaram, Swihart, & DeWoody, 2016). To effectively use molecular techniques to make inference to underlying ecological processes, it is necessary to evaluate and account for the various levels of uncertainty in observation processes (Bohmann et al., 2014; Hunter et al., 2015; McClintock et al., 2010; Spear, Groves, Williams, & Waits, 2015). Because the sources of error for genetic methods span multiple levels of biological organization, the overall error structure should be hierarchical—that is total error should be equivalent to a series of conditional probabilities.

Detection rates of individuals in the field may be influenced by vegetation or weather conditions such as dense foliage or rain that create visual obstructions, or road or stream noise that cause auditory disturbance, or time of day or season that may cause behavioral differences in detection rates (Christy, Yackel Adams, Rodda, Savidge, & Tyrrell, 2010; Farnsworth et al., 2002). Observation processes for molecular tools are unique because there are additional levels of observation (e.g., availability of DNA, DNA capture rate, and amplification success). Environmental conditions (pH, temperature, substrate, etc.) may strongly influence the observation processes at these different levels. Incorrectly accounting for multi‐level influences could lead to biases in estimates of the underlying ecological process (Gu & Swihart, 2004). It is possible to get unbiased estimates of the cumulative detection process without accounting for the different levels of uncertainty (Schmidt, Kéry, Ursenbacher, Hyman, & Collins, 2013). However, identifying the observational processes by level allows for the optimization of sampling effort to increase overall detection probability. Thus, an understanding of both the factors affecting observation as well as the level they act on is critical for accurately quantifying ecological processes such as species distribution (invasive or endangered) or pathogen spread using molecular methods such as environmental DNA (eDNA; DNA collected from the environment rather than directly from a target species).

Detection of a target species’ eDNA from water bodies is emerging as a potentially valuable method to infer the distribution of species and pathogens (Bohmann et al., 2014; Hunter et al., 2015; Takahara, Minamoto, & Doi, 2013). Efforts have been largely focused on aquatic and semi‐aquatic species such as amphibians (Biggs et al., 2015; Pilliod, Goldberg, Arkle, & Waits, 2013; Schmidt et al., 2013), reptiles (Hunter et al., 2015; Piaggio et al., 2014), invertebrates (Doi et al., 2017; Thomsen et al., 2012), and fish (Takahara et al., 2013; Thomsen et al., 2012). As more recent work has applied eDNA detections in water bodies to terrestrial mammals (Rodgers & Mock, 2015; Ushio et al., 2017; Williams, Huyvaert, & Piaggio, 2017), the method could be especially useful for detecting new invasions of terrestrial species in the early stages. To maximize the utility of eDNA in understanding landscape‐level ecological processes (e.g., occupancy, distribution), it is important to determine the observation processes (i.e., detection rates) at many levels (Willoughby et al., 2016) that may influence the probability of detecting DNA in the environment. Once cells are shed, abiotic and biotic factors begin to degrade DNA (Barnes & Turner, 2016). Previous work has shown that the influence of microbial communities, temperature, pH, UV, and other environmental factors will impact the availability of DNA in the environment (Barnes & Turner, 2016) and therefore should be accounted for when assessing the detection probability of eDNA.

Occupancy models (MacKenzie et al., 2006) are well suited for quantifying species distributions in space and time while accounting for levels of uncertainty across observation processes, and have been used to assess species presence through eDNA (Hunter et al., 2015; Schmelzle & Kinziger, 2016; Schmidt et al., 2013; Valentini et al., 2016). When using eDNA as a passive detection method, there is the added complexity over a classical occupancy model because, even when the species of interest is present, the DNA in a given sample may not be present (Furlan, Gleeson, Hardy, & Duncan, 2016; Williams et al., 2017). Therefore, the detection process (*p*) of a species by eDNA can be split into three levels: the probability that DNA is present and can be detected, “available”, in the water sample (*θ*), the probability of capturing DNA in an extraction procedure (*γ*) given it is available in the sample, and the probability of amplifying DNA in a qPCR run (*δ*) given it has been captured in an extract. Separating these probabilities allows evaluation of factors that influence observation of DNA across each of these levels as well as their influence on overall detection probability. By separating the observation process out in this manner, we can identify the level that will improve the most by increased sampling and thus ensure resources are optimally allocated. Enhancing overall detection probability will result in better inference of underlying ecological processes using this approach for the detection of cryptic (Bickford et al., 2007) or elusive (Rogala et al., 2011) species.

We sampled for eDNA of wild pigs (*Sus scrofa*), an invasive terrestrial species in North America and other parts of the world. They are capable of rapid geographic expansion (Snow, Jarzyna, & VerCauteren, 2017) and cause high levels of damage to ecosystems and the economy (Anderson, Slootmaker, Harper, Holderieath, & Shwiff, 2016; Chavarria, Lopez, Bowser, & Silvy, 2007; West, Cooper, & Armstrong, 2009). Because they are reliant on water bodies for drinking and wallowing, and their distribution and densities vary widely, they are a good model system for evaluating the potential application of eDNA for assessing the presence of terrestrial species, understanding spatial expansion of invasive species, and developing protocols for monitoring the effectiveness of invasive species control programs. As elimination programs for wild pigs occur in many countries across the globe, there is great need for cost‐effective methods for evaluating success and guiding decisions (Hone, 1983; Korn & Bomford, 1996; Saunders & Bryant, 1988). Further, in areas without wild pigs, the ability to evaluate reports of sightings or sign is critical to implementation of early control measures that could curb the establishment of a newly invasive population. Environmental DNA is a promising tool to aid in these monitoring efforts because it has the potential to be an efficient field method (Williams et al., 2017). Yet, application of eDNA in terrestrial species is currently limited by a poor understanding of the observation processes and inferences of ecological processes.

Our objectives were to: (a) examine factors that influence observation processes across several levels: availability of eDNA in water sampling, the capture rate of DNA in the extraction process, and the amplification probability during qPCR, (b) evaluate our ability to correctly assess target species presence at sampling sites given our observation process, and (c) develop an adaptive approach to eDNA collection and analysis to balance field and laboratory effort for efficiency. By accounting for multiple levels of uncertainty in the observation process, we aim to improve estimation of ecological processes and provide guidance for future sampling designs using eDNA for detection of a target species.

## 2 METHODS

### 2.1 Study area

One way to understand the detection probability of a method, such as eDNA, is to evaluate the ability to detect a target species in a setting where the species is known to be present. Typically, the presence or absence of a species is of primary interest and the detection probability is often thought of as a nuisance parameter that must be accounted for to obtain unbiased estimates of species occupancy (MacKenzie et al., 2002). However, to get precise estimates of detection probability based on the detection method alone, we can reframe the problem to eliminate the “nuisance” parameter of occupancy probability (MacKenzie et al., 2006) by sampling in an area where the presence of the species is known. This allows for the assessment of factors that might influence detection such as environmental (abiotic and biotic) factors or laboratory processes.

**Figure 1:**
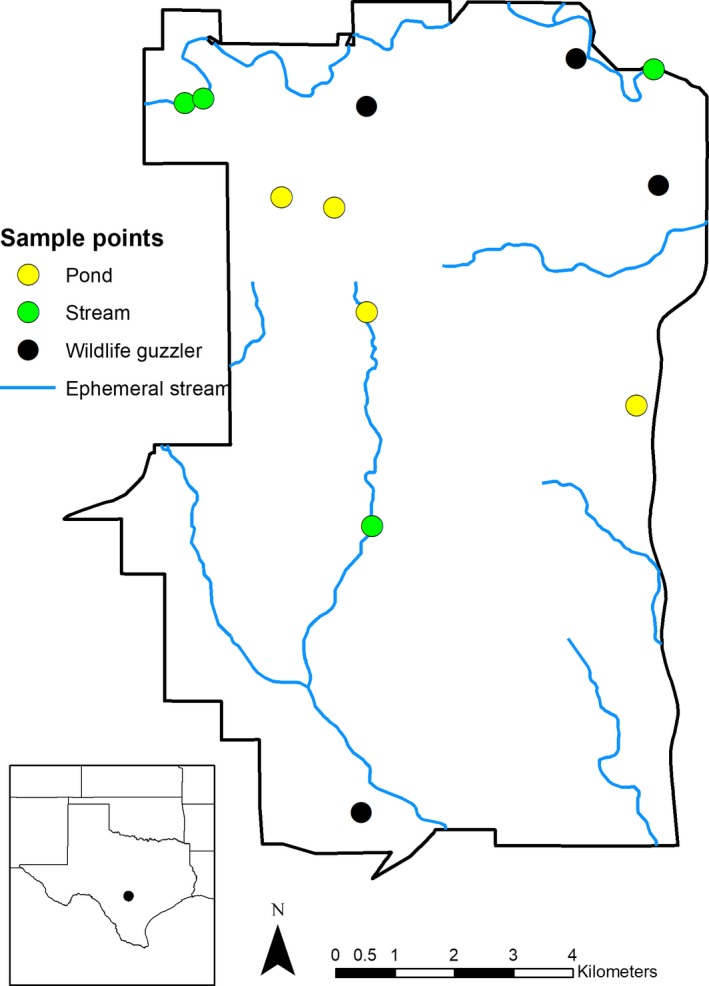
Map of study area, Camp Bullis, Texas. Sampling locations are shown as colored circles (pond‐yellow, stream‐green, and wildlife guzzler‐black)

Our study was conducted at Camp Bullis Training Site (112.9 km^2^), in northern Bexar County, Texas, USA operated by Joint Base San Antonio (Figure 1). Camp Bullis is a restricted access property with perimeter fencing and high densities of pigs. This property is located in the Edwards Plateau ecoregion of the south‐central semi‐arid prairies of Texas (Bailey, 1980, 1998). Vegetation is primarily comprised of an oak woodland and grassland matrix (Wills, 2006). Topography consists of rolling hills with limestone outcrops, rocky soils, and caves typical of the Edwards Plateau (Kastning, 1983). Semi‐ephemeral streams and pools fluctuate throughout the year, usually peaking during the wettest month of May. Camp Bullis reports that >140 guzzlers are scattered throughout the property as catchments of rainwater for wildlife, although not all of these guzzlers are maintained and hold water.

### 2.2 Cameras

Remote cameras (Reconyx^®^ PC900, Holmen, WI, USA) were mounted on trees overlooking the focal sampling sites (water bodies) where obvious sign of animal visitation had occurred (e.g., tracks, trails, or scat). Cameras were mounted ≤10 m from the water and were programed to record motion‐activated images. Upon motion, the cameras took three photos that were 30 s apart, followed by a quiet period of 15 min. The memory cards and batteries of cameras were refreshed once per month, and the camera positioning adjusted depending on water level. From the camera trap data located at each water body at the time of sampling, we recorded: the number of hours since the last pig visit to the water body; the number of images in the last day, week, and month with pigs; and the average group size of pigs in pictures with pigs using the Colorado Parks and Wildlife Photo Database (v3.0) for image processing (Ivan & Newkirk, 2016). These data were collected to help us assess our ability to successfully collect wild pig eDNA after a documented visitation to the site.

### 2.3 Field eDNA collection

Previous studies some of us developed (Williams, Huyvaert, & Piaggio, 2016; Williams, Huyvaert, Vercauteren, Davis, & Piaggio, 2018; Williams et al., 2017) a method for detecting pig DNA in water. Typically, wild pigs use smaller bodies of water or edges of water bodies. The activity of pigs and the nature of the water bodies they use mean that the target water bodies are often very turbid with high degree of suspended sediment and floating debris. Thus, we specifically tested methods for capturing DNA from such systems (Williams et al., 2017). Further, to meet our agency's goal of detecting wild pigs, we had some practical considerations to build into our method development: (a) our field personnel could not transport extra equipment such as filters, (b) field personnel did not have time to conduct filtering of samples in the field, (c) field samples could not require a cold chain for field preservation, and (d) samples collected had to be made small and light. Thus, the collection implemented in this study reflects these practical considerations as incorporated into the optimized collection and lab assay we developed for wild pig detection (Williams et al., 2016, 2017, 2018).

We selected 12 sites within Camp Bullis for water sample collection (Figure 1). We stratified the sites by three water body types (natural ponds, streams, and wildlife guzzlers). We randomly selected four sites from each of three water body types: natural ponds, stream (broken into 100 m segments), and wildlife guzzlers. Water samples were collected once a month for 2 months (September and October 2016). We focused on this time period to avoid camera damage (i.e., March–May) associated with spring flooding and because our access was restricted to some of the sites during hunting season (i.e., November–December).

Each sampling event consisted of taking 10 water samples from each of 12 sites. Sampling was evenly spaced in wildlife guzzlers, and spread around ponds and along streams. The goal of taking multiple samples was to get a diverse selection of water from the site to maximize the chance that the samples would contain wild pig DNA and overcome the heterogeneous distribution of eDNA in the environment (Furlan et al., 2016). Samples were collected by submerging a 60 ml Nalgene bottle ten centimeters below the surface of the water (when possible) until the bottle was filled to a line marked on the bottle at 45 ml. Then, 15 ml of Longmire's lysis buffer was added to the sample (1 part Longmire's: 3 parts sample water as in Williams et al., 2016). With the collection of each sample, our intent was to collect as little sediment as possible to avoid colloidally bound DNA that may not have been shed recently (Barnes et al., 2014). A negative control (15 ml tap water carried by sampler with 5 ml of Longmires buffer added) was collected during each sampling session at each site. Gloves were worn at all times while sampling and were changed between sites. Collectors were instructed not to walk in the water body to avoid contamination between sites. Each bottle was labeled with a unique ID relating to site, field replicate number, and date collected. The locality information, number of samples, type of water body (i.e., wallow, moving, artificial waterer/tank, other), approximation of size of water body (i.e., small (<10 m^2^), medium (10–1,000 m^2^), large (>1,000 m^2^)), pH, approximation of depth where the sample was collected (cm), if it was collected along a transect or randomly, and whether there was evidence of pig activity in the area (i.e., tracks, rooting, wallowing) were all recorded as site‐level characteristics. Samples were stored in a box at ambient temperature until being shipped to the United State Department of Agriculture (USDA) Animal and Plant Health Inspection Service (APHIS) National Wildlife Research Center (NWRC) within a week of collection. Once received at NWRC, the samples were placed in a −80°C freezer until further processing.

### 2.4 eDNA capture, extraction, and amplification

**Figure 2:**
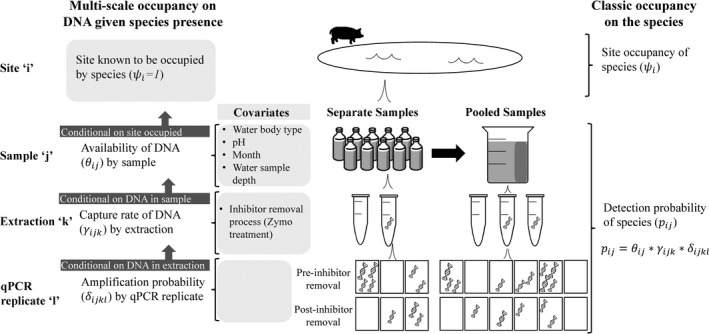
Schematic showing the different levels of uncertainty. On the right hand side is the classic two‐level occupancy model structure (MacKenzie et al., 2002) with the occupancy of the species at the site level (*ψ*_*i*_) and the overall detection probability by sample “*j*” at site “*i*” (*p*_*ij*_). On the left, the detection probability is split into different levels (similar to: Schmidt et al., 2013): availability of DNA by sample (*θ*_*ij*_) given pigs are at the site, capture rate of DNA by extraction (*γ*_*ijk*_) given DNA was available in the sample, and amplification probability by qPCR replicate (*δ*_*ijkl*_) given DNA was captured in the extraction. Covariates are shown by the level they are modeled on

We compared two strategies for our extraction and amplification procedures. For the first procedure, we followed protocols that recommend pooling samples by site (Biggs et al., 2015) and conducting three extractions from the pooled sample (Piaggio et al., 2014). From each extraction, we ran five qPCR replicates before inhibitor removal and 5 qPCR replicates after inhibitor removal (Figure 2; details on inhibitor removal below). This method was more efficient for laboratory work; however, it produced few positive water samples despite camera data showing pigs at sampled water bodies. We suspected that pooling all 10 samples from each site diluted the DNA below detectable levels. Therefore, for our second strategy, we examined the 10 samples by site separately and conducted two extractions per sample. From each extraction, we ran three qPCR replicates before inhibitor removal and three qPCR replicates after inhibitor removal (Figure 2). Therefore, each individual sample was split in half, with one half being pooled by site and the second half being treated separately.

The analyses of the separate water samples by site increased the detection probability substantially (see results) but proved burdensome for laboratory personnel and resources. Therefore, for the second month (October), we compared the amplification for pooled samples as in the previous month, but we additionally used a removal sampling approach on the separate samples to reduce laboratory costs. For the removal sampling approach, we analyzed one water sample at a time (conducting two extractions per sample and three qPCR replicates per extraction), per site and stopped once a positive detection occurred. For each sample, we proceeded as for the standard sampling design in which we took two extractions per sample and ran six qPCR replicates per extraction (three pre‐inhibitor removal and three post‐inhibitor removal).

Each field sample for both months had a total volume of 60 ml (45 ml sample water + 15 ml Longmires = 60 ml). Therefore, 30 ml from each of the field samples were pooled and 30 ml were kept separate. Each extraction for both pooled and separate samples was conducted on 15 ml subsamples. We conducted three extractions of 15mls from our pooled water samples and two extractions of 15 ml for our separate samples.

We followed an optimized eDNA extraction protocol for detecting wild pig DNA in turbid waters (Williams et al., 2017). Briefly, this involved centrifuging each 15 ml subsample at 9,000 g for 15 min at room temperature, extracting DNA from the pellet with the DNeasy mericon Food Kit (Qiagen) in triplicate. We included a negative control in each set of extractions to monitor for contamination.

The number of qPCR replicates varied by month for pooled samples (first month of study: five replicates, second and third month of study: six replicates) and were conducted on a CFX96 (BioRad) following Williams et al. (2017). Each qPCR reaction was a 30 μl reaction. Each reaction contained 15 μl Taqman environmental mastermix (Life Technology), 1 μl BSA, 6 μl distilled water, 1 μl of each primer (10 μmol/L), 1 μl of the probe (2.5 μmol/L), and 5 μl of template DNA. Amplificarions were performed on a Biorad real‐time PCR thermocycler (Biorad, Hercules, CA, USA). The real‐time thermocycling was a single cycle for 10 min at 95°C, then 50 cycles of 95°C for 15 s, and a final extension of 1 min at 52°C. A standard curve was run in triplicate with each run using dilutions of a synthetic sequence of our target amplicon (gBlocks^®^ Gene Fragments, IDT). We included three negative controls with each qPCR reaction and also attempted amplification from extraction and field‐collected negative controls to monitor for contamination.

For analysis of each of the 10 samples per site (no pooling), we performed two extractions per sample (15 ml each) for September and October with three qPCR technical replicates per extraction. Further, each extraction, from both pooled and individual water samples, was amplified with qPCR before and after OneStep Inhibitor Removal kit (Zymo Research) to determine the influence of removing inhibitors. All instruments and bench tops were decontaminated after each run with 10% bleach and all steps associated with pre‐PCR, PCR, and post‐PCR were conducted in different laboratory rooms, each dedicated to the processing of low quality/quantity DNA. Our assay was sensitive enough to detect down to 1 copy/μl of DNA (LOD—limit of detection). DNA extracts may be heterogeneous in the distribution of DNA and since only 5 μl out of the 150 μl elution of extract is used, we may not have transferred enough template DNA to be successfully amplified successfully. Inhibitors are humic substances that may be coextracted with DNA and interfere with downstream processing (PCR), and are therefore can affect the probability that PCR will be successful (Matheson, Gurney, Esau, & Lehto, 2010; McKee, Spear, & Pierson, 2015).

### 2.5 Analytical methods

To estimate the presence of wild pig DNA at each level of sampling, we adopted the multi‐scale occupancy framework developed by Nichols et al. (2008) and was first proposed for use with eDNA by Schmidt et al. (2013) to include multiple levels in the observation process to the classic occupancy model (Figure 2; MacKenzie et al., 2002, 2006). When using eDNA to detect a species, we considered the following levels of the observation process (Figure 2): (a) the sampling process level which describes the probability that DNA of the study species is available for detection at the sample level, *j,* given that the species is present at site *i* (*θ*_*ij*_), (b) the capture process level which describes the probability DNA is captured at the extraction level, *k*, given DNA is in sample *j* (*γ*_*ijk*_), and (c) the amplification process level which describes probability of amplification in a qPCR assay, *l*, of the sample given DNA is captured in extraction *k* (*δ*_*ijkl*_; Kendall & White, 2009; Schmidt et al., 2013). The multi‐level occupancy model can be written as a series of Bernoulli random variables such that *z*_*i*_ represents the true presence/absence status of the species of interest at site *i*;* a*_*ij*_ is the availability status of the DNA in sample *j* given the species is present at site *i*;* d*_*ijk*_ is the capture status of DNA in extraction *k*, of sample *j*, at site *i*; and *y*_*ijkl*_ is the amplification status of the qPCR assay, *l*, given DNA is present at site *i,* available in sample *j*, and captured in extraction *k*.

(1) $$z_{i}∼\text{Bernoulli}\left(\psi_{i}\right)$$

(2) $$a_{\mathrm{ij}}|z_{i}∼\text{Bernoulli}\left(z_{i}\theta_{\mathrm{ij}}\right)$$

(3) $$d_{\mathrm{ijk}}|a_{\mathrm{ij}}∼\text{Bernoulli}\left(a_{\mathrm{ij}}\gamma_{\mathrm{ijk}}\right)$$

(4) $$y_{\mathrm{ijkl}}|d_{\mathrm{ijk}}∼\text{Bernoulli}\left(d_{\mathrm{ijk}}\delta_{\mathrm{ijkl}}\right)$$

Since we knew the true occupancy status at all of our sites was 1 (confirmed by camera data and pig sign at all sites), we fixed the top level ($\psi_{i}=1$) and conducted a three‐level multi‐scale occupancy analysis (Nichols et al., 2008) to parse out the variability associated at the levels of water samples, extractions, and qPCR replicates. Detection data include only detections (*y* = 1) and non‐detections (*y* = 0). To estimate each parameter in the three level model, we need replication at each level in the hierarchy (*θ*_*ij*_ *, γ*_*ijk*_, *δ*_*ijkl*_). We only had replicates at each level in the months where we kept the water samples separate, thus only data from months 2 and 3 were used in this analysis.

For the multi‐scale analysis, we examined the potential relationship of DNA availability and the pH of the water, the water body type (i.e., wildlife guzzler, pond, and stream), and the water sample depth (all of these were site‐level characteristics; Figure 2). Additionally, we allowed availability to vary by month. The only factor we examined on the capture rate by extraction was the inhibitor removal treatment. In addition, we had several covariates from the camera data that we thought might predict DNA availability by sample including: hours since last pig visited the water body, the number of pictures with pigs within the last month, and the average group size when pigs visited the water body. We examined DNA availability both with and without the camera data. The camera data were used to provide a better understanding of the biological processes that relate to DNA availability.

We used the three level multi‐scale occupancy model to understand which factors influence the observation processes at different levels, satisfying our first objective. By better understanding the observation process, we should be better able to evaluate the biological state of interest (i.e., occupancy of the species). Our second objective was to determine whether we could accurately assess the status of animal presence in an area with known wild pig populations. Therefore, we used a standard two‐level occupancy model (classic occupancy) where the detection parameter (*p*_*ij*_ here) is the product of the DNA availability probability by sample (*θ*_ij_), the capture probability by extraction (*γ*_ijk_), and the DNA amplification probability (*δ*_ijkl_); the species detection status is given by *w*_*ij*_. We used the information gained regarding important covariates from the three‐level, multi‐scale analysis to inform the detection process in this analysis.

For the classical occupancy analysis, we condensed the detection data such that if any qPCR replicate in any extraction had a detection, the detection history would be a 1, otherwise, it would be a 0. We used the pre‐ and post‐ inhibitor removal treatment periods as our two detection occasions. We used this approach to compare occupancy estimates between pooled and separate samples and among months.

Our third objective was to evaluate the power to correctly detect animal presence under different conditions and provide guidance on eDNA sampling design that would be most reliable and efficient in the field and laboratory. We used estimates from the multi‐scale occupancy analysis (where *ψ* = 1). These provided us with realistic estimates of the probability of detecting wild pigs at different water body types, under different water conditions, and pre‐ and post‐ inhibitor removal treatment. We ignored information from the cameras as this type of data would not be available in most eDNA based field studies. To estimate the cumulative probability of detection (denoted with an “*”), given sampling effort (number of samples/extractions/replicates, *n*) for the availability of DNA by sample (*θ*), capture probability of DNA through extraction (*γ*), and the amplification probability of qPCR (*δ*), we used equation (6). The variance for the cumulative probability was calculated using the delta method (equation (7); Powell, 2007). We used the cumulative probabilities to determine the minimum number of samples per site, extraction replicates, and qPCR replicates to achieve 90% detection probabilities at those respective levels. We used the cumulative probabilities of detection to determine the minimum number of samples per site, extraction replicates per sample, and qPCR replicates per extraction to achieve 90% detection probability for each level of the observation process/observation hierarchy. Using equations 5 and 6, we calculated the cumulative probability of detection given sampling effort for different ranges of effort (Supporting Information Appendix S1).

(5) $$p_{\mathrm{ij}}=\theta_{\mathrm{ij}}*\gamma_{\mathrm{ijk}}*\delta_{\mathrm{ijkl}}$$

(6) $$x^{*}=1-{\left(1-x\right)}^{n}$$

(7) $$\text{var}\left(x^{*}\right)=n^{2}{\left(1-x\right)}^{2*(n-1)}\text{var}\left(x\right)$$

We analyzed all data using occupancy models in Program MARK (Cooch & White, 2016; White & Burnham, 1999). We used the single‐season, multi‐scale occupancy option (Nichols et al., 2008) for the multi‐scale analysis. We used the standard occupancy estimation method for the classic occupancy analysis. For both analyses, we treated the sites across months independently as we were interested in the independent assessments for each month to determine if there was a temporal component to the estimates of occupancy, and because the estimation of extinction and colonization parameters would be extraneous nuisance parameters. Model comparison was conducted using Akaike's Information Criterion corrected for small samples sizes (AICc, Burnham & Anderson, 2002; Doherty, White, & Burnham, 2012). For the multi‐scale occupancy model investigating relationships with availability of DNA and qPCR amplification success, we examined all additive combinations of covariates (including the inhibitor removal on capture rate and pH, month, water body type, water depth, pictures of pigs per month, average group size per picture, and hours since last visit on availability). The cumulative covariate weights, covariate weights >0.5 were considered to be important (Doherty et al., 2012). When model uncertainty (multiple models within 2 ΔAICc of the top model) existed, we used model averaging to estimate occupancy and detection rates (Burnham & Anderson, 2002).

## 3 RESULTS

**Figure 3:**
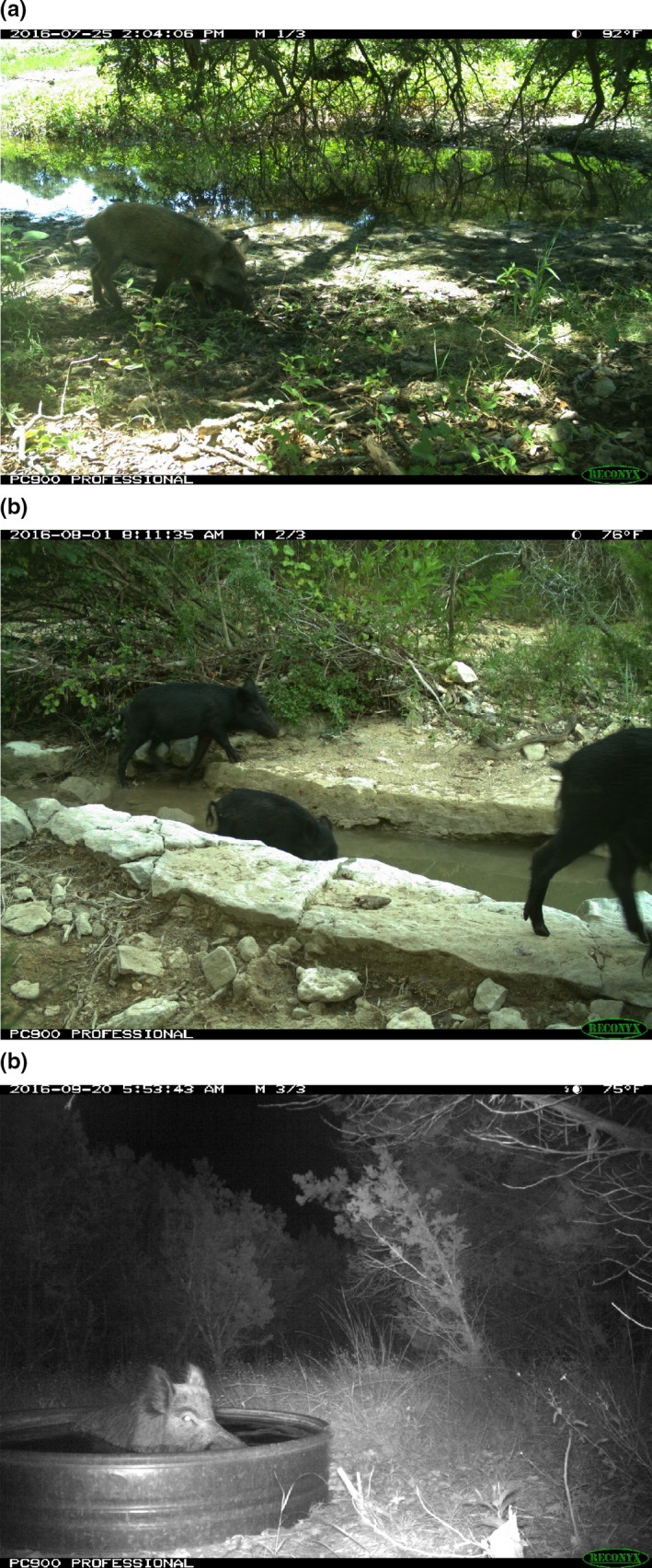
Example images of wild pigs at pond sites (a), stream sites (b), and wildlife guzzler sites (c). Images are from camera traps located at eDNA collection sites on Camp Bullis, TX

In month one, one stream site was dry and was not sampled. All sites were sampled in month two. During the study, there were 12,842 photographs taken from the 12 cameras (one at each site). There were 1,003 photographs of wild pigs (264 at pond sites, 428 at stream sites, and 311 at wildlife guzzler sites; examples shown in Figure 3). For the laboratory analyses, we had both positive and negative controls in all reactions and at all steps and we did not get amplification in any of our negative controls.

**Figure 4:**
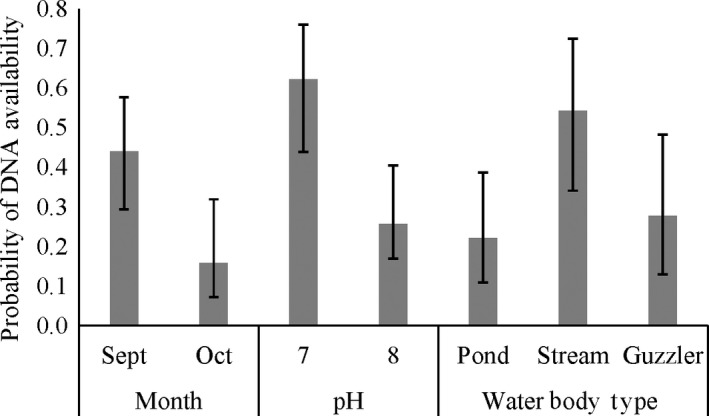
Relationships of covariates with the probability of DNA availability at the sample level (*θ*_ij_). Average probability of availability from the model containing important covariates (top model) by month with 95% confidence interval. The relationship between pH of the water body and the DNA availability with 95% confidence intervals. The relationship between water body type and eDNA availability with 95% CIs. Each estimate per covariate (month, pH, and water body type) is given as an average across other covariates

The results from the multi‐scale occupancy analysis (objective 1) showed that DNA availability at the individual sample level was influenced by the month in which the sampling was conducted and the pH and the type of water body (Table 1, Supporting Information Appendix S3). September had a higher availability of DNA than October (*β* = 1.31, *SE* = 0.05; Figure 4). The availability of DNA was higher in sites with pH values closer to 7 than 8 (*β* = −1.65, *SE* = 0.52; Figure 4). The average pH value in our study was 7.6 (*SD* = 0.47; Supporting Information Appendix S2). DNA availability tended to be higher for stream samples than for ponds or wildlife guzzlers, however, the 95% confidence intervals overlapped (*β*_guzzler_ = −1.18, *SE* = 0.60; *β*_pond_ = −1.34, *SE* = 0.55; Figure 4). We examined a linear trend with pH but demonstrate the estimated availability associated with two values of pH to avoid extrapolation past of this trend outside the range of data we examined (Figure 4). The depth of the water sample was not strongly related to DNA availability (Table 1). Sample depths averaged 8.6 cm (*SD* = 2.8, Supporting Information Appendix S2). Confidence intervals for point estimates in figures rely on the asymptotic normality of maximum likelihood estimates and are back transformed from the logit link estimates and standard error.

**Table 1:** Cumulative covariate weights from multi‐scale occupancy model selection procedure comparing relative covariate relationships between DNA availability at the sample level (*θ*) and capture rate at the extraction level (*γ*) from eDNA samples at Camp Bullis, TX Sept–Oct 2016. Covariates are separated by parameter (availability or detection). Covariate weights are shown for models including and excluding camera data

Covariate by parameter	Cumulative covariate weight	
	Excluding camera data	Including camera data
Availability (*θ*)		
pH	0.98	0.98
Month	0.92	0.97
Pictures per month	—	0.95
Group size	—	0.42
Water body type	0.80	0.23
Hours since last visit	—	0.30
Water sample depth	0.35	0.28
Capture rate (*γ*)		
Inhibitor removal	0.31	0.31

**Figure 5:**
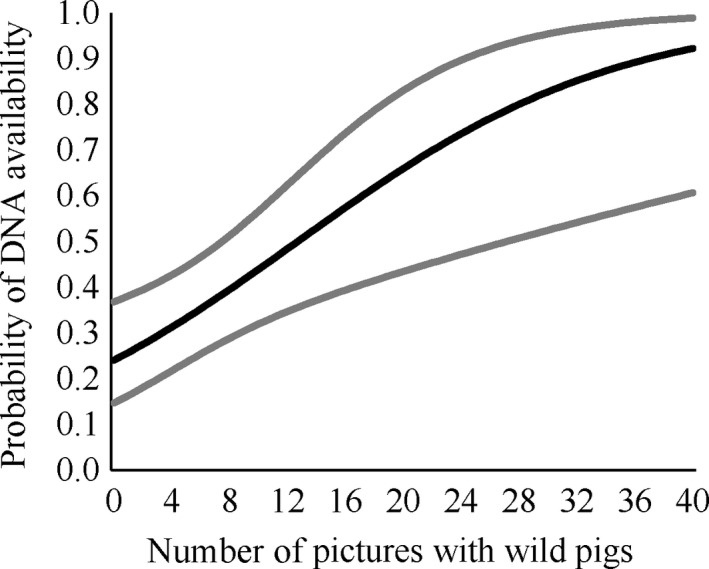
The relationship between DNA availability and the number of pictures with pigs in the month prior to sampling and the DNA availability with 95% confidence intervals

When camera data were included, the month of sampling and pH was still important predictors of availability but water body type was not (Table 1, Supporting Information Appendix S4). Instead, the number of photographs with pigs within the last month was related to DNA availability (*β* = 0.09, *SE* = 0.03; Table 1) and was substantially higher when more photographs with wild pigs were observed within the last month (Figure 5). The average number of photographs with pigs in the last month was 25.5 (*SD* = 49.8, Supporting Information Appendix S2). Neither of the other covariates from the camera data was strongly related to DNA availability. The average number of hours since the last pig detection was 319 (*SD* = 434, Supporting Information Appendix S2), and the average group size in photographs was 1.48 (*SD* = 0.37, Supporting Information Appendix S2).

The impact of the inhibitor removal treatment did not have a substantial effect on capture rate by extraction (*β *= −0.16 *SE* 0.34). The estimated capture rate without treatment was 0.49 (*SE* 0.07), whereas the estimated capture rate with treatment was 0.43 (*SE* 0.07). However, there were several instances where wild pig DNA was only captured without the inhibitor removal treatment and several instances where wild pig DNA was only captured from extractions with the inhibitor removal treatment. Therefore, even though capture rates were similar either before or after the inhibitor removal treatment, the combined capture rates including both captures before and after the inhibitor removal did increase the overall apparent detection (raw samples without treatment that had a detection = 0.6; raw samples with treatment had a detection = 0.5, raw samples with or without treatment that had a detection = 0.79). No covariates were examined with reference to the qPCR amplification rate. The estimated amplification rate was 0.38 (*SE* 0.04).

**Figure 6:**
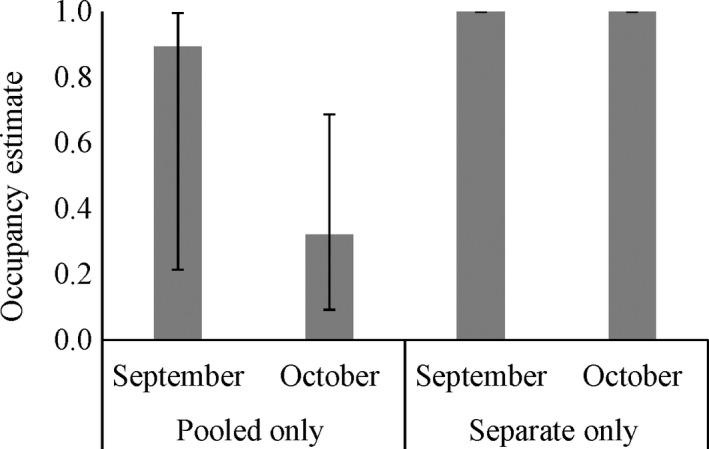
Occupancy estimates (with 95% confidence intervals) of wild pigs from eDNA samples at sites at Camp Bullis, TX Sept–Oct 2016. Estimates by month are shown for pooled versus separate samples

To determine if we could correctly detect pig presence (objective 2), we used the important covariates from the previous analysis as covariates on detection (*p*) to estimate overall occupancy (excluding camera data as they would not be present in a true field study). When only the pooled data were considered, the occupancy estimates were biased low for all months, but the estimates were highest in September (Figure 6). Occupancy was correctly estimated to be 1 when separate samples per site we examined for both the standard sampling design (all 10 water samples were analyzed, month 2) and for the removal sampling design (processing samples ceased once a detection was found by sample, month 3; Figure 6). For comparison, we also examined how the estimates in September may have changed had we used a removal sampling approach and not the full data set. We found that occupancy was estimated to be the same as when the standard sampling design was used, but the detection probability was lower (*p* = 0.22, *SE* = 0.09 for removal sampling compared to *p* = 0.29, *SE* = 0.07 for standard sampling).

**Figure 7:**
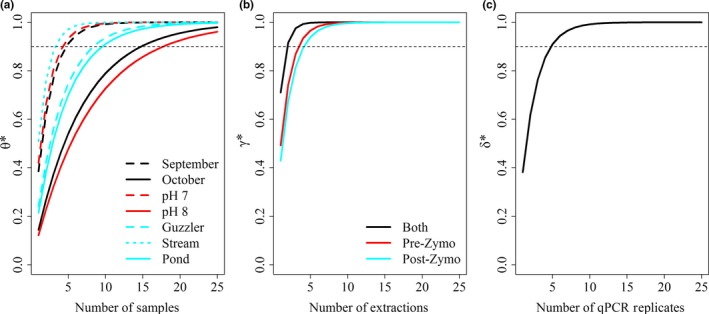
Cumulative probabilities given a hypothetical number of replicates for three levels of the multi‐scale analysis on detection using eDNA. The cumulative detection probability is the product of the different levels of uncertainty (Supporting Information Appendix S1). The cumulative availability of DNA (θ*, the probability that DNA is observed in at least one sample given the site is occupied) by water sample (a) is shown by the number of separate water samples collected based on estimates in September (dashed black line) and October (solid black line), for water pH values of 7 (dashed red line) and 8 (solid red line), and sampling from wildlife guzzlers (dashed blue line), streams (dotted blue line), and ponds (solid blue line). (b) The cumulative capture probability (γ*, the probability that DNA is observed in at least one extraction given it is available in the sample) of DNA by number of extractions is shown for estimates based on pre‐inhibitor removal treatment (solid red line), post‐inhibitor removal treatment (solid blue line), and both pre‐ and post‐ inhibitor removal treatment (solid black line). (c) The cumulative amplification probability (δ*, probability that DNA is observed in at least one qPCR replicate given it is captured in the extraction) is shown by the number of qPCR replicates (solid black line). An interactive version can be found in the supplemental information

We used the probabilities of DNA availability under each of the conditions (month, pH, water body type), while holding the other values constant at their mean, to examine the cumulative probability of availability under different numbers of water samples. To achieve a mean 90% cumulative availability probability in September, we only would have needed five separate samples (95% CI: 3–8 samples), whereas in October we would have needed 15 (95% CI: 5–25+ samples; Figure 7, using equations 6 and 7). Water pH had a strong influence the number of water samples as when pH is 7 we would need five samples (95% CI: 3–7 samples) to ensure at least one detection but if pH was 8 we would need 18 samples (95% CI: 6–25+ samples) per site. The influence of water body type demonstrated that wildlife guzzlers and ponds required 9 (95% CI: 4–14 samples) and 10 (95% CI: 5–15 samples) samples per site, respectively, but streams would only need four samples (95% CI: 2–5 samples) for 90% cumulative probability of availability. The cumulative capture probability at the extraction level was slightly higher without inhibitor removal than with this treatment, requiring only four extractions per sample (95% CI: 3–5) compared to five (95% CI: 3–6) to achieve a 90% cumulative capture probability. Conducting qPCR amplifications on both types of samples (with and without inhibitor removal) showed a substantial improvement on DNA capture rates (Figure 7) requiring only two extractions to achieve 90% cumulative probability (95% CI 2–3 samples). Five qPCR replicates per extraction were required to achieve a 90% cumulative amplification probability.

## 4 DISCUSSION

Understanding the observation process is an important first step for quantifying species distribution processes, especially for practical purposes in low‐density populations (i.e., early detection of invasive species or timely detection of endangered species). When detection does not occur, it does not confirm that the species is absent—there is the possibility that the species is present but that detection probability was below 100% due to variability in observation processes (Schmidt et al., 2013). Our results showed that for observation processes that span multiple levels of uncertainty (e.g., non‐invasive detection methods such as eDNA), it is important to understand the role of different factors across these levels and which environmental factors may have the strongest influence on ecological inference. Dissecting the observation process not only allowed for better inference of ecological processes but also provided a platform for planning sampling adaptively, in a way that minimizes laboratory costs and time while maximizing species detection probability.

We expected differences in detection probabilities of wild pig eDNA (*p*) among water body types due to variations in pig behavior and visitation rates, and abiotic conditions that affect DNA. Detection rates were similar between wildlife guzzlers and ponds, which might retain DNA more similarly when compared to streams. We expected streams to have a lower detection rate assuming that the movement of water would relate to a lower retention rate of DNA. However, streams had the highest detection rates of all water bodies in our study while ponds and guzzlers had similarly lower detection rates. Our study was conducted between the seasonal end of the summer and the beginning of the fall, when water levels were lower and thus streams were intermittent with pooling and low flow. These areas of multiple pools along a streambed proved conducive for DNA retention and detection, perhaps due to their attractiveness to pigs for multiple wallowing sites in proximity to one another (suggested by photographic evidence).

The probability of detecting DNA is likely affected by water quality (Barnes & Turner, 2016; Barnes et al., 2014). If sampling was conducted when water resources were plentiful, detection probability would have been lower as the DNA would have been more diluted. The probability of availability of DNA was highest in September when temperatures in the area were higher with less rainfall (water was scarcer) than in October when detection rates were lower. If sampling were conducted during cooler times of year, when there is more standing water, the detection rates would likely have been lower than we observed. Lower detection rates would mean that more field samples would need to be collected, and thus when designing an eDNA monitoring method seasonally varying detection rates will be important to incorporate in practice.

Consistent with previous studies, we found pH to be a strong indicator of DNA availability in water samples. Lorenz and Wackernagel (1987) found that DNA has a higher rate of adsorption to sand, and thus is more available, when the pH is neutral (pH 7). Additionally, DNA is known to degrade more rapidly or adsorb to certain soil particle types when pH deviates from neutral (Barnes et al., 2014; Lorenz & Wackernagel, 1987). In particular, Strickler, Fremier, and Goldberg (2015) and Seymour et al. (2018) found that degradation rates were higher in more acidic conditions, and degradation rates were lower or similar in alkaline compared to neutral conditions (respectively). Interestingly, we found higher detection rates in neutral conditions compared to slightly alkaline conditions. The fact that pH had such a strong relationship with DNA availability was surprising given the range we observed was narrow (7–8.4). Considerably, more samples would need to be collected to ensure a cumulative availability rate >90% when the pH of the water body was close to 8 compared to 7 (18 samples would be required compared to 5). In addition, pH may also be correlated with other factors we did not measure that influence wild pig behavior (e.g., plant community composition, turbidity of the water). Regardless, it appears that pH is an important factor in the probability of detecting eDNA and the variability in observation processes using this method. Thus, if pH is not appropriately accounted for across geographic sites and seasons, species distributions based on eDNA could be biased.

We found the observation processes (detection probability) of wild pig from eDNA to be variable during our study. One possible explanation for that variability is that the behavior of wild pigs in the area may influence the detection probability. We used cameras to examine how detection related to pig behavior. It was surprising that the time period since camera detection was not strongly related to DNA availability given that wild pig eDNA degrades with time (Williams et al., 2018). Despite the fact that degradation rates are higher for a single wild pig relative to a group (Williams et al., 2018), group size also did not strongly correlate with DNA availability. However, the number of pictures of wild pigs visiting our sampled water sites was strongly related to detection probability, suggesting that the general level of wild pig activity influenced detection. Thus, higher level of use may act similarly to larger group sizes in terms of DNA degradation rates. Since we are particularly interested in detecting pigs at low densities using eDNA, our actual detection probability may be lower than calculated in this study when implemented for detections of wild pigs in newly invaded areas or areas where control has reduced the population to low densities. Therefore, larger number of samples should be taken in the field to offset the lower levels of DNA availability that may occur.

Replicates that do not provide the same detection (some are positive, and some are negative) may arise from qPCR instrumentation variability when at the lower limits of DNA quantity (Hunter et al., 2017). The assay we applied for detection of wild pig eDNA has a limit of detection of 10 copies/μl (>95% of 8 qPCR replicates of our standard curve amplified; Bustin et al., 2009). Thus, one level of variability in our observation process involves the detection probability of low quantity/quality DNA. We accounted for this by including amplification probability with qPCR in our model and considering any positive as a detection (assuming no false positives). This is a risk if the specificity of our test has the possibility for false positives. We tested for false positives due to cross‐reactivity and found high specificity in our assay for wild pigs (Williams et al., 2017). Issues with specificity would lead to incorrectly declaring species present when it is absent (Type I error) and issues with sensitivity would lead to declaring a species absent when it is present (Type II error). Generally, with early detection for an invasive species, the risk of declaring a non‐detection when the species is present (low detection probability or poor sensitivity) is a considerably worse type of error than poor specificity due to the potential damage that may occur if an invasion went undetected.

We were particularly focused on the issues of false negatives (which may be very costly in our case), and we were confident that false positives were not an issue in our study. However, there are some cases where the cost of responding to a new invasion may be considerable and thus there would be substantial concern for declaring a species is present when it is not (false positive). To that end, some studies require that two or more PCR replicates confirm a detection before they will be declared a positive (Kriger, Hero, & Ashton, 2006). If false positives are possible at the PCR level, then modifications must be made to the occupancy analysis as that is a clear violation of the occupancy assumptions (Mackenzie, 2005). There are several advances to occupancy modeling that will accommodate false positives (Miller et al., 2011; Royle & Link, 2006) and approaches that have been incorporated into eDNA analyses (Ficetola, Taberlet, & Coissac, 2016; Lahoz‐Monfort, Guillera‐Arroita, & Tingley, 2016).

Pooling field‐collected eDNA samples by site and taking extractions from subsamples of the pooled sample (Biggs et al., 2015; Piaggio et al., 2014) reduces laboratory costs over running multiple samples per site separately. Replicate samples per site are collected because the DNA distribution in water is heterogeneous (Furlan et al., 2016), such that some samples may not contain DNA. Pooling the field samples eliminates the ability to have true replicates to estimate the detection process. The subsampling of the pooled samples only allows for pseudo‐replicates. Our results demonstrated that pooling field replicates greatly reduced the detection probability and thus added to the variability in observation processes when compared to analyzing the replicate samples separately. This suggests that although DNA may be present in the pooled samples, the act of pooling may increase the effect of dilution (perhaps requiring extra filtration) and the use of pseudo‐replicates reduces our power to make inference on the hidden biological state of interest. Therefore, if pooled samples are used, a species may go undetected. For threatened or endangered species, this may result in failure to provide conservation measures, whereas for an invasive species a non‐detection could lead to a new invasion progressing unchecked and allowing it to become established.

Although keeping samples per site separate greatly increased our detection probability, it also greatly increased the laboratory time and expenses. Instead of three extractions for each site, we conducted 20 extractions per site (2 per each of 10 field water samples). This method is likely prohibitively laborious and costly for use as a standard sampling procedure to detect invasive terrestrial mammals. Therefore, we adapted our approach to a removal design (by sample), which has been found to be the most efficient design for occupancy studies (MacKenzie & Royle, 2005). However, MacKenzie and Royle (2005) point out that removal designs are less robust to violations of assumptions compared to a standard design. For example, occupancy should be constant during the sample frame (if a site is occupied it should be occupied during the entire sampling period), detection rates will be biased low if violations of this assumption occur, but the bias will be greater for the removal design than the standard design. This suggests that the maximum number of samples needed for a removal design might be more than the total number of samples for a standard design. Based on our estimated detection probability of 0.2 and our high occupancy rate, MacKenzie and Royle (2005) would recommend 23 samples be collected per site. Fortunately, the field costs of collecting additional water samples per site are marginal and extractions would only need to be conducted on additional samples if no detections were made from each previous extraction.

Our field collection protocols were strategically designed to streamline and simplify sampling so they could be used by a variety of agencies in a variety of field conditions (Williams et al., 2016). Our methods are species‐specific—we focused on small water bodies which wild pigs are likely to use for drinking and wallowing to optimize detection of this terrestrial species. To come up with a generic method to sample these small, turbid water bodies, we needed to use smaller water samples than are often used for detecting aquatic species (e.g., Furlan et al., 2016; Pilliod et al., 2013; Spear et al., 2015). The smaller water volumes used in our study may contribute to the lower levels of detection that we observed compared to other studies. Our results suggest that the probability of detection would be increased by collecting more water samples per site (which would increase the overall volume of water collected per site). By collecting more samples, we will still be able to use the same protocol for sample collection (which helps with consistency across study areas) while addressing the issue that small water volume may have on detection probability.

The field costs to collect additional samples may be low, but processing those samples in the laboratory is not. The removal approach may reduce overall costs. However, if no detections occur then the costs will be the same as the standard sampling approach if all samples are analyzed. Although costs in this case may be high, eDNA has been found to be a cost‐effective option for monitoring populations of aquatic species compared to trapping (Lugg, Griffiths, van Rooyen, Weeks, & Tingley, 2018) and may be found to be worth the costs when compared to potential costs of undetected invasions of invasive species.

Pooling samples per site may be used as a first step in a removal sampling approach, and if there is no detection, testing the field samples could proceed discretely and in succession. However, using the data from September, if we had used the pooled data as a first step in a removal sampling approach (3 extractions and 12 qPCR replicates) and then continued with the separate samples (two extractions and six qPCR replicates) until we came to a detection, we would have needed a total of 228 qPCR replicates to analyze all of the data. If we had forgone the pooled samples and just conducted the removal approach on the separate field samples, we would have needed only 84 qPCR replicates to analyze all the data. Thus to detect rare individuals, we recommend a removal approach, with eDNA analysis on the separate samples, to optimize detection probabilities and reduce false negatives and variability in observation processes. A removal approach is also advantageous as collecting field samples are relatively inexpensive relative to the requisite cost of associated lab work; so this method reduces costs while not compromising power.

Using the detection probabilities of wild pig eDNA (*p*) estimated from the separate samples per site, we calculated the cumulative detection probability given the number of samples analyzed through qPCR to evaluate cumulative detection rates of using more samples than we collected, similar to Schmidt et al. (2013). Because availability varied by month, pH, and water body type, the number of samples necessary per site to have a high cumulative availability probability (θ*, where *θ* is plugged into equation 8, and “*n*” is the number of samples) was dependent on site conditions. For example, in a stream sampled in September, we had over a 90% cumulative availability probability with only three samples analyzed, but in streams in October we needed to analyze at least six samples to have the same cumulative availability probability. While in wildlife guzzlers or ponds in September, we needed to analyze at least five samples for the same level of confidence and in October, we did not reach a 90% cumulative availability probability until 16 samples were analyzed. Thus by collecting a potentially superfluous number of water samples in the field, future studies will be better equipped to handle issues of poor DNA availability that may occur due to study timing, water body type, or other environmental conditions that reduce overall detection probability.

We conducted qPCR both pre‐ and post‐ inhibitor removal treatment. The treatment strips away inhibitors that prevent qPCR from amplifying DNA. However, in cases where there is a low quantity of DNA in the sample, the inhibitor removal treatment may result in reducing the DNA and not just the inhibitors. This might suggest that it is the number of extractions that is important and not the inhibitor removal process. However, we found many instances of detections that only happened pre‐ treatment and some that only happened post‐ treatment. Additionally, DNA concentrations of extracts before inhibitor removal treatment were low. Although, conducting one extraction with data both pre‐ and post‐ inhibitor removal resulted in a similar cumulative DNA capture rate to conducting two extractions with data either just pre‐inhibitor removal or just post‐inhibitor removal, we recommend that all samples are subject to qPCR amplifications both with and without inhibitor removal to maximize detection probability of target DNA or that an internal positive control (IPC) be incorporated into the qPCR assay to monitor inhibition (Goldberg et al., 2016).

## 5 CONCLUSIONS

We found that observation processes of an invasive terrestrial mammal using eDNA are variable and dependent on the conditions of the water body sampled and laboratory processes. We recommend collecting a minimum of 10 water samples per site, but 20 samples would be better, and using a removal approach to laboratory analysis; more samples will be needed when the pH of the water body is not neutral. The adaptive analysis process will maximize detection while making efficient use of resources. We recommend running qPCR both pre‐ and post‐ inhibitor removal to increase capture rate of DNA. Variation in time of year and water body type will also impact the overall detection probability using eDNA and must be accounted for or estimates of species presence will be biased which could have serious implications for conservation or invasive species management. As has become part of best practices for non‐invasive genetics studies (another approach for utilizing low quality/quantity DNA available in the absence of the target species), we strongly advocate for conducting field studies on the system of interest to determine the factors influencing the study site and the target species (Lonsinger et al., 2015; Taberlet, Waits, & Luikart, 1999; Waits & Paetkau, 2005). Using multi‐scale occupancy modeling for inference of species distributions will provide more robust inferences about factors affecting the observation processes of eDNA, which is particularly important for designing efficient sampling protocols. The multi‐scale analysis may not need to be continued once greater understanding of the different levels of uncertainty are reached; however, new programs and packages (Dorazio & Erickson, 2017; Hunter et al., 2015; White & Burnham, 1999) have been developed that make performing this type of analysis marginally more work than a standard occupancy approach and therefore may be worth continuing. Improving the understanding of the observational process will help provide better understanding of the ecological processes influencing the distribution of the target species and guidance for future research and management.

## AUTHOR CONTRIBUTIONS

AJD, KEW, NPS, AJP, and KMP designed the research. KEW and NPS performed the research. AJD analyzed the data. AJD, KEW, NPS, AJP, and KMP wrote the paper.

## DATA ACCESSIBILITY

Detection data and related covariates are archived in Dryad data repository (https://doi.org/10.5061/dryad.1fp259b).
