# Supplementary material for: Accounting for observation processes across multiple levels of uncertainty improves inference of species distributions and guides adaptive sampling of environmental DNA
Source: Ecol Evol. 2018 Oct 23;8(22):10879–92. doi: 10.1002/ece3.4552 (PMC6262734; doi:10.1002/ece3.4552)
Supplement: Supplementary file 1 [file ECE3-8-10879-s001.pdf]

Supplemental Information for:

Accounting for observation processes across multiple levels of uncertainty improves inference of species distributions and guides adaptive sampling of environmental DNA

Amy J. Davis, Kelly E. Williams, Nathan P. Snow, Kim M. Pepin, and Antoinette J. Piaggio

Table of Contents:

**Appendix A: Detection probability application**	Page 1
**Appendix B: Covariate ranges**	Page 2
**Appendix C: AICc table without camera data**	Page 3
**Appendix D: AICc table with camera data**	Page 4

Appendix A. Application to look at potential cumulative detection probabilities (p) given estimated DNA availability rates (θ), capture rates (γ), and amplification rates (δ) under different conditions from environmental DNA of wild pigs, collected from water samples in Texas in 2016.
https://ajdavis.shinyapps.io/eDNA_app/

Appendix B. Covariate ranges by water body type for eDNA collection sites on Camp Bullis, TX. The box plots show the mean value across sites and month by water body type (black line). The box edges are the first and third quartile range, the lines show the 95% interquartile range, and the dots are outliers.

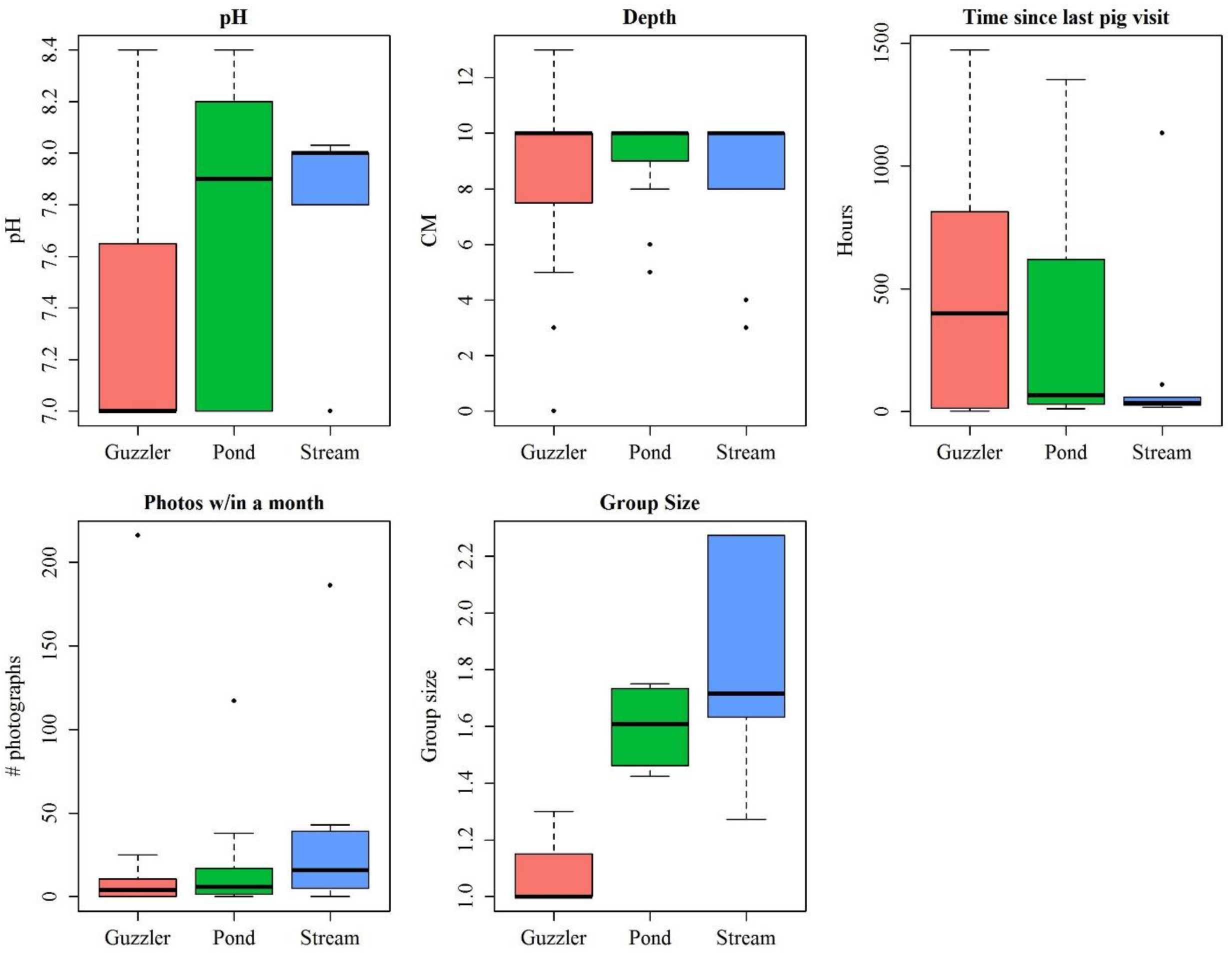

Appendix C: AICc table without camera data

Table of model results for availability and capture rate examination for wild pig occupancy estimation from Camp Bullis, TX from Sept-Oct 2016. These results do not include camera data.

Model	AICc	Delta AICc	AICc Weights	Num. Par	Deviance
Psi(Month[a] + Type[b] + pH) p(.)	337.33	0.00	0.39	6	324.82
Psi(Month + Type + pH) p(Zymo[c])	339.31	1.98	0.15	7	324.62
Psi(Month + Type + pH + Depth[d]) p(.)	339.49	2.16	0.13	7	324.80
Psi(Month + pH) p(.)	341.46	4.13	0.05	4	333.22
Psi(Month + Type + pH + Depth) p(Zymo)	341.49	4.17	0.05	8	324.60
Psi(Month + pH + Depth) p(.)	341.85	4.52	0.04	5	331.49
Psi(Type + pH) p(.)	343.17	5.84	0.02	5	332.80
Psi(Month + pH) p(Zymo)	343.38	6.05	0.02	5	333.02
Psi(Type + Depth) p(.)	343.46	6.14	0.02	5	333.10
Psi(Type + pH + Depth) p(.)	343.50	6.17	0.02	6	330.99
Psi(Month + Type + Depth) p(.)	343.71	6.38	0.02	6	331.20
Psi(Month + pH + Depth) p(Zymo)	343.81	6.48	0.02	6	331.29
Psi(pH + Depth) p(.)	344.18	6.85	0.01	4	335.94
Psi(Month + Depth) p(.)	344.98	7.66	0.01	4	336.74
Psi(Type + pH) p(Zymo)	345.18	7.85	0.01	6	332.67
Psi(Type + Depth) p(Zymo)	345.48	8.15	0.01	6	332.97
Psi(Type + pH + Depth) p(Zymo)	345.53	8.20	0.01	7	330.84
Psi(Depth) p(.)	345.71	8.38	0.01	3	339.57
Psi(Month + Type + Depth) p(Zymo)	345.73	8.40	0.01	7	331.04
Psi(Month + Type) p(.)	346.01	8.69	0.01	5	335.65
Psi(pH + Depth) p(.)	346.14	8.81	0.00	5	335.77
Psi(pH) p(.)	346.52	9.19	0.00	3	340.37
Psi(Month + Depth) p(Zymo)	346.94	9.61	0.00	5	336.57
Psi(Depth) p(Zymo)	347.66	10.33	0.00	4	339.42
Psi(Month + Type) p(Zymo)	348.01	10.68	0.00	6	335.50
Psi(Month) p(.)	348.34	11.01	0.00	3	342.20
Psi(pH) p(Zymo)	348.45	11.12	0.00	4	340.21
Psi(Type) p(.)	350.02	12.69	0.00	4	341.78
Psi(Month) p(Zymo)	350.26	12.94	0.00	4	342.02
Psi(Type) p(.)	352.04	14.72	0.00	5	341.68
Psi(.) p(.)	352.43	15.10	0.00	2	348.36
Psi(.) p(Zymo)	354.37	17.05	0.00	3	348.23

[a] Month = indicator for a difference between months

[b] Type = Water body type indicator

[c] Zymo = Inhibitor removal indicator

[d] Depth = Water sample depth (cm)

Appendix D: AICc table with camera data

Table of model results for availability and capture rate examination for wild pig occupancy estimation from Camp Bullis, TX from Sept-Oct 2016. These results do include camera information. These results show all models that are within 5 delta AICc values, the null model, and the fully parameterized model for comparison.

Model	AICc	Delta AICc	AICc Weights	Num. Par	Deviance
Psi(Month[a] + pH + PMth[b] + GrpSize[c]) p(.)	329.15	0	0.13	6	316.64
Psi(Month + pH + HrsVd + PMth + GrpSize) p(.)	329.15	0.00	0.13	7	314.47
Psi(Month + Type[e] + pH + PMth + GrpSize) p(.)	329.21	0.06	0.13	8	312.32
Psi(Month + pH + PMth + GrpSize) p(Zymo[f])	331.09	1.94	0.05	7	316.40
Psi(Month + pH + HrsV + PMth + GrpSize) p(Zymo)	331.12	1.97	0.05	8	314.23
Psi(Month + Type + pH + PMth + GrpSize) p(Zymo)	331.20	2.06	0.05	9	312.09
Psi(Month + Type + pH + HrsV + PMth + GrpSize) p(.)	331.28	2.13	0.04	9	312.16
Psi(Month + pH + Depth[g] + PMth + GrpSize) p(.)	331.32	2.18	0.04	7	316.64
Psi(Month + pH + Depth + HrsV + PMth + GrpSize) p(.)	331.34	2.19	0.04	8	314.45
Psi(Month + Type + pH + Depth + PMth + GrpSize) p(.)	331.43	2.28	0.04	9	312.31
Psi(Type + pH + Depth + HrsV + PMth + GrpS + HrsC + Month2) p(Zymo)	334.07	4.92	0.01	12	308.09
Psi(.) p(.)	352.43	23.28	0	2	348.36

[a] Month = indicator for a difference between months

[b] PMth = Number of pictures with pigs per month

[c] GrpSize = Average number of pigs in pictures with pigs

[d] HrsV = Hours since the last pig visit before sampling based on photographs

[e] Type = Water body type indicator

[f] Zymo = Inhibitor removal indicator

[g] Depth = Water sample depth (cm)
